# Benefits of glioma resection in the corpus callosum

**DOI:** 10.1038/s41598-020-73928-x

**Published:** 2020-10-06

**Authors:** Marie-Therese Forster, Marion Behrens, Irina Lortz, Nadine Conradi, Christian Senft, Martin Voss, Maximilian Rauch, Volker Seifert

**Affiliations:** 1 Department of Neurosurgery, Goethe University Hospital, Schleusenweg 2-16, 60528 Frankfurt am Main, Germany; 2 University Cancer Center Frankfurt (UCT), Goethe University Hospital, Theodor Stern Kai 7, 60590 Frankfurt am Main, Germany; 3 Department of Neurology, Goethe University Hospital, Schleusenweg 2-16, 60528 Frankfurt am Main, Germany; 4 Dr. Senckenberg Institute of Neurooncology, Goethe University Hospital, Schleusenweg 2-16, 60528 Frankfurt am Main, Germany; 5 Department of Neuroradiology, Goethe University Hospital, Schleusenweg 2-16, 60528 Frankfurt am Main, Germany

**Keywords:** Oncology, Signs and symptoms

## Abstract

Due to anticipated postoperative neuropsychological sequelae, patients with gliomas infiltrating the corpus callosum rarely undergo tumor resection and mostly present in a poor neurological state. We aimed at investigating the benefit of glioma resection in the corpus callosum, hypothesizing neuropsychological deficits were mainly caused by tumor presence. Between 01/2017 and 1/2020, 21 patients who underwent glioma resection in the corpus callosum were prospectively enrolled into this study. Neuropsychological function was assessed preoperatively, before discharge and after 6 months. Gross total tumor resection was possible in 15 patients, and in 6 patients subtotal tumor resection with a tumor reduction of 97.7% could be achieved. During a median observation time of 12.6 months 9 patients died from glioblastoma after a median of 17 months. Preoperatively, all cognitive domains were affected in up to two thirds of patients, who presented a median KPS of 100% (range 60–100%). After surgery, the proportion of impaired patients increased in all neurocognitive domains. Most interestingly, after 6 months, significantly fewer patients showed impairments in attention, executive functioning, memory and depression, which are domains considered crucial for everyday functionality. Thus, the results of our study strongly support our hypothesis that in patients with gliomas infiltrating the corpus callosum the benefit of tumor resection might outweigh morbidity.

## Introduction

Despite recent advances in molecular diagnostics and modern individual treatment, most patients suffering from glioma still face limited survival. Broad evidence exists that besides age, the preoperative Karnofsky performance score and adjuvant radiochemotherapy, the extent of surgical tumor resection (EoR) has a significant impact on patients’ survival. Nevertheless, patients suffering from gliomas that invade the corpus callosum rarely undergo surgical tumor resection. On the one hand, these tumors are considered more aggressive due to corpus callosum invasion and thus, to dissemination to both hemispheres, on the other hand, many neurosurgeons refrain from tumor resection within the corpus callosum in order to spare patients’ neurological or neurocognitive functions.

The corpus callosum is the largest interhemispheric commissure providing a connection between homologous cortical areas. There is ongoing debate whether it exerts an excitatory or inhibitory function, although evidence substantiating the theory of excitation predominates^1^. Thus, enforced integration of cerebral processing between the two hemispheres is attributed to the corpus callosum, supported by the observation of increased corpus callosum size in persons capable of completing complex tasks and in persons with less behavioral laterality^2,3^. Only recently, left-brain oriented persons, being thought to be more lateralized due to the frequent ipsilateral location of language, have been shown to have a significantly smaller corpus callosum than right-brain oriented persons^4^.

Lesioning the corpus callosum may produce various symptoms subsumed under the callosal disconnection syndrome^5,6^. Left hand tactile agnosia, alexia in the left visual field and all above impairment of behavior are among the most common symptoms. Alexithymia, difficulty of attention, impaired verbal and working memory and difficulties in learning and coordinating new bimanual tasks can be detected applying neuropsychological tests in patients with damaged corpus callosum.

We observed many patients with huge frontal glioma and corpus callosum invasion who presented with avolition, aboulia or impaired consciousness already at the time of diagnosis, and many of these patients died within a few months after tumor biopsy and despite radiochemotherapy. This led us to hypothesize that tumor resection might not harm patients more than refraining from it. Likewise, removal of tumor tissue invading the corpus callosum could even improve patients’ neurological condition, assuming that reducing the mass effect and modifying the microenvironment of the surrounding neurons would translate into better neurocognitive and neurological function^7,8^.

Interestingly, only recently some publications focused on the resection of butterfly glioblastomas or tumors involving the corpus callosum. Chaichana et al. reported on significant shorter overall survival for patients suffering from butterfly glioblastoma compared to patients with glioblastoma in other regions^9^. However, median survival of patients with butterfly glioblastoma was markedly improved by tumor resection, doubling a patient’s remaining life time in comparison to tumor biopsy only; a resection of ≥ 65% of the tumor volume was found to be associated with longer overall survival. Another analysis on the impact of EoR in butterfly glioblastoma was provided by Dayani et al., and they found out that the amount of total tumor reduction, but not EoR within the corpus callosum influenced patients’ overall survival (OS)^10^. Chen et al. equally observed EoR to be independently associated with longer OS in both, low- and high-grade glioma, whereas no such association was found for corpus callosum involvement which had no significant impact on OS^11^.

While these three publications provided evidence that the resection of tumors involving the corpus callosum improves patients’ overall survival^9–11^, only one publication by Dziurzynski et al. reported on the doubtful benefit of the resection of butterfly glioblastomas^12^. However, adjuvant treatment had been administered to barely half of their patients, probably confounding their results.

As a consequence, we decided to perform tumor resection in a cohort of patients suffering from glioma invading the corpus callosum and to evaluate prospectively the impact of surgery on patients’ clinical and neuropsychological function and OS. We hypothesized that removal of tumors with corpus callosum invasion might improve patients’ prognosis while maintaining or even ameliorating their neurological function, thereby breaking a passed-on dogma of refraining from tumor resection within the corpus callosum.

## Methods

### Design

We performed a prospective single-center study in patients who underwent surgical resection of tumors invading the corpus callosum. Patients underwent neuropsychological evaluation as part of their pre-surgical work-up, before their hospital discharge as well as after a follow up period of at least 3 months between January 2017 and January 2020.

Study approval was granted by the ethics committee of Frankfurt University Hospital (approval number SNO-09-2017). All procedures performed were in accordance with the ethical standards of the institutional research committee and with the standards laid down in the Declaration of Helsinki. All patients gave written informed consent prior to data collection.

### Patients

Between 01/ 2017 and 01/ 2020, all patients meeting the criteria for study inclusion were identified. Inclusion criteria comprised (1) adult patients aged ≥ 18 years, (2) radiological suspicion of glioma with corpus callosum invasion, (3) indication for tumor resection and (4) fluent knowledge of German. The indication for surgical treatment was evaluated in a multidisciplinary tumor-board for each patient. Study-specific exclusion criteria were (1) any previous brain tumor treatment except tumor biopsy, (2) tumor spread into basal ganglia precluding tumor removal at a great extent, (3) lack of adequate social or family support needed for adherence to the further postoperative therapeutic regimen, and (4) any medical contra-indication for brain tumor surgery.

Clinical characteristics, including the Karnofsky-Performance Score, results of magnetic resonance imaging and data on tumor characteristics including molecular analysis of tumor tissue were recorded prospectively for every patient.

### Imaging and volumetric assessment of EOR

An additional review of tumor location, extent of lesion resection and response assessment in neuro-oncology (RANO) criteria was conducted by a trained neuro-radiologist before data analysis. In detail, structural MRI including standard sequences (T1-weighted (w), T2-w, T2*-w, FLAIR, diffusion-weighted imaging (DWI), T1-w with contrast agent) and a 3-dimensional T1-w sequence with contrast agent was performed prior to surgery, early postoperatively (i.e., within 72 h following surgery) as well as thereafter every 3 months. Tumor volume was assessed on 3D-T1-w sequences using Brainlab software (Brainlab AG, Munich, Germany) by manual segmentation. EoR was calculated as the difference of pre- and early postoperative tumor volumes, expressed as percentage. Gross tumor resection was defined as complete tumor resection, and subtotal and partial tumor resection were defined as 90–99% and < 90% EoR, respectively.

### Neurocognitive assessment and test battery

Neurocognitive assessment was performed at three different time points; as part of the preoperative work-up (t1), at the end of hospital stay (t2) and after a median follow-up-period of 5.3 (range 3.1–12.8) months (t3).

All patients underwent neurocognitive testing before surgery. Data on postoperative assessment had to remain incomplete either due to patients’ non-compliance, due to refusal to undergo further evaluation or because of death in one patient. Thus, follow-up evaluation at time points t2 and t3 was possible in a subset of 16 patients and 11 patients, respectively. All neurocognitive evaluations were performed by the same trained clinical neuropsychologist. Each assessment took place under optimal testing conditions and took patients approximately 1 h to complete. The applied elaborated test-battery included tests for attention, language, executive functioning, memory, visuospatial functioning, object recognition and bimanual coordination, the mini-mental state examination (MMSE) for assessing orientation, as well as the assessment of emotion such as anxiety and depression by the Hospital Anxiety and Depression Scale (HADS). A detailed list of all tests is provided in Table 1.

**Table 1:** Tasks per neurocognitive domain.

Cognitive domain	Tasks
Attention	Trail Making Test, Part A and Part B (TMT-A and TMT-B)^30^
Language	Boston Naming Test (BNT)^31^
Executive functioning	Regensburg Word Fluency Test (RWT)^32^
	Trail Making Test Switching ratio (TMT-B/A)^14^
	Verbal Learning and Memory Test (VLMT)^33^—Subtest Intrusion
Memory	Verbal Learning and Memory Test (VLMT)^33^—subtests total
	Recall, Delayed Recall, Delayed Recognition
Figurative memory	Pentagon Drawing Test—delayed recall^34^
Visuospatial functioning	Pentagon Drawing Test—copy^34^
Bimanual coordination	Alternating Opening and Closing of Fists
	Alternating Pronation and Supination
	Luria Sequence^35^
	Finger Tapping Sequence
Object recognition	Tactile Object Recognition^36^
	Tactile Letter Recognition^36^
Orientation	Mini Mental Status Examination^37^
Depression and anxiety	Hospital Anxiety and Depression Scale (HADS)^38^

### Statistical analysis

Patients’ performances for the domains attention, language, executive functioning and memory were compared to published normative data of healthy controls accounting for age, gender, and educational level. The according scores of each test were then transformed into Z-scores, and cognitive decline was defined as a Z-score of less than − 1.5 SD^13,14^. Orientation, visuospatial functioning, object recognition and bimanual coordination were considered impaired as soon as one error per test occurred. Thereafter, both, the Z-score for each domain and the percentage of patients impaired per domain were calculated for each time point.

For analyzing the statistical effect of surgery on neurocognition (i.e. t2 vs. t1 and t3 vs. t1) we applied a Chi-Square test for each neurocognitive domain. In all evaluations, a *p* value of ≤ 0.05 was considered statistically significant.

### Ethics approval

The ethics committee of Frankfurt University Hospital approved the protocol and conduction of this study (SNO-09–2017).

### Consent to participate

All patients gave written informed consent on the evaluation of their clinical and neurocognitive data.

## Results

### Patients characteristics

**Figure 1:**
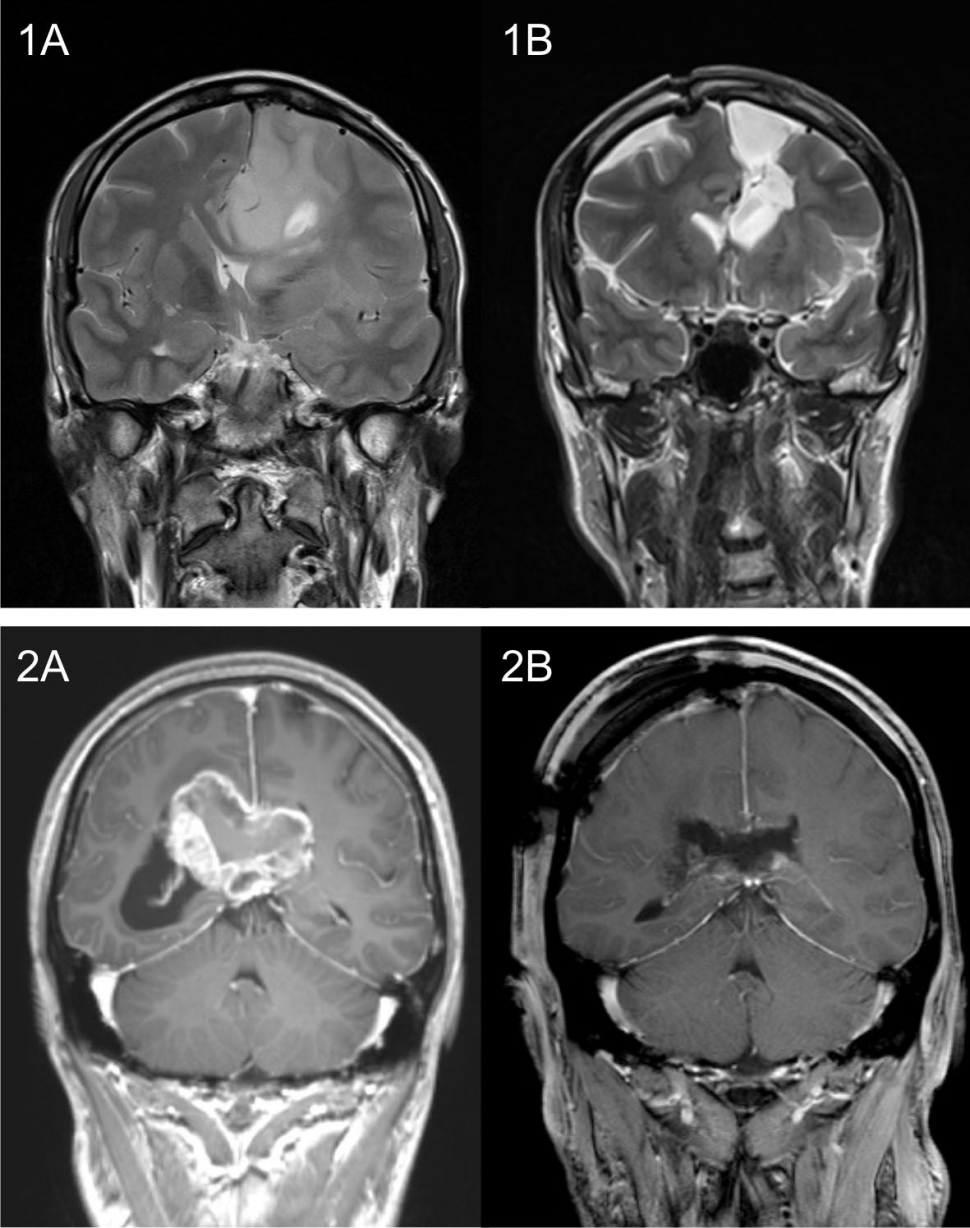
Examples of 2 patients with glioblastomas invading the corpus callosum. (**1A)** and (**2A)** depicting the tumor prior to surgery and (**1B)** and (**2B)** showing postoperative results. Patient 1, 36-years old, underwent STR of huge left frontal glioblastoma, followed by radiochemotherapy. He didn’t show any severe cognitive impairment at any time point (i.e. neither prior to surgery nor at long-term follow-up). Patient 2, 38-years old, underwent STR of huge glioblastoma involving the posterior body of the corpus callosum. Prior to surgery, he suffered from severe impairment of memory, improving gradually thereafter, as did postoperative new mild deficits in attention and language.

Baseline characteristics are presented in Table 2. In total, the study cohort included 21 patients (12 females, 9 males) aged 41.1 (range 23.9–76.0) years, in whom surgical procedures were performed between January 2017 and January 2020. Tumor location was frontal in 20 patients and parietal in 1 patient, with corpus callosum invasion of different degrees, including 2 huge butterfly gliomas and further 6 tumors involving both hemispheres, and with additional involvement of the cingulate gyrus in 11 patients. Prior to surgery, median tumor volume came up to 66.6 cm^3^. Tumor resection was complete in 15 patients, and subtotal in 6 patients, with median residual tumor volume of 2.1 cm^3^ and median EoR reaching 97.7% in these 6 patients (Fig. 1). Histopathology revealed glioblastoma WHO grade IV in 17 patients, oligodendroglioma WHO grade III in 2 patients, and anaplastic astrocytoma WHO grade III and diffuse astrocytoma WHO grade II in one patient each.

**Table 2:** Baseline and surgical characteristics.

Characteristics	Number (percentage) or median (min–max)
Gender, female	12 (57%)
Age, years	41.1 (23.9–76.0)
**Histology**	
Astrocytoma	2 (9.5%)
Oligodendroglioma	2 (9.5%)
Glioblastoma	17 (81%)
**WHO grade**	
II	1 (4.7%)
III	3 (14.3%)
IV	17 (81%)
IDH mutation	9 (42.9%)
Bilateral involvement	8 (38.1%)
Predominant laterality, right	11 (52.4%)
Involvement of cingulate gyrus	11 (52.4%)
KPS at diagnosis, %	100 (60–100)
Preoperative tumor volume, cm^3^	66.6 (1–143)
**EoR**	
100% (GTR)	15 (71.4%)
90–99% (STR)	6 (28.6%)
< 90% (PR)	0
**Adjuvant treatment**	
Combined radiochemotherapy	17 (81%)
Radiotherapy	1 (4.7%)

Adjuvant tumor treatment was recommended for all but one patient, however, one patient refused further treatment, and one patient who had undergone subtotal resection suffered from early tumor progression during rehabilitation therapy precluding her from any further treatment. Thus, 17 patients were treated with concurrent radiochemotherapy and one patient received radiotherapy alone.

### Clinical and survival outcomes

**Figure 2:**
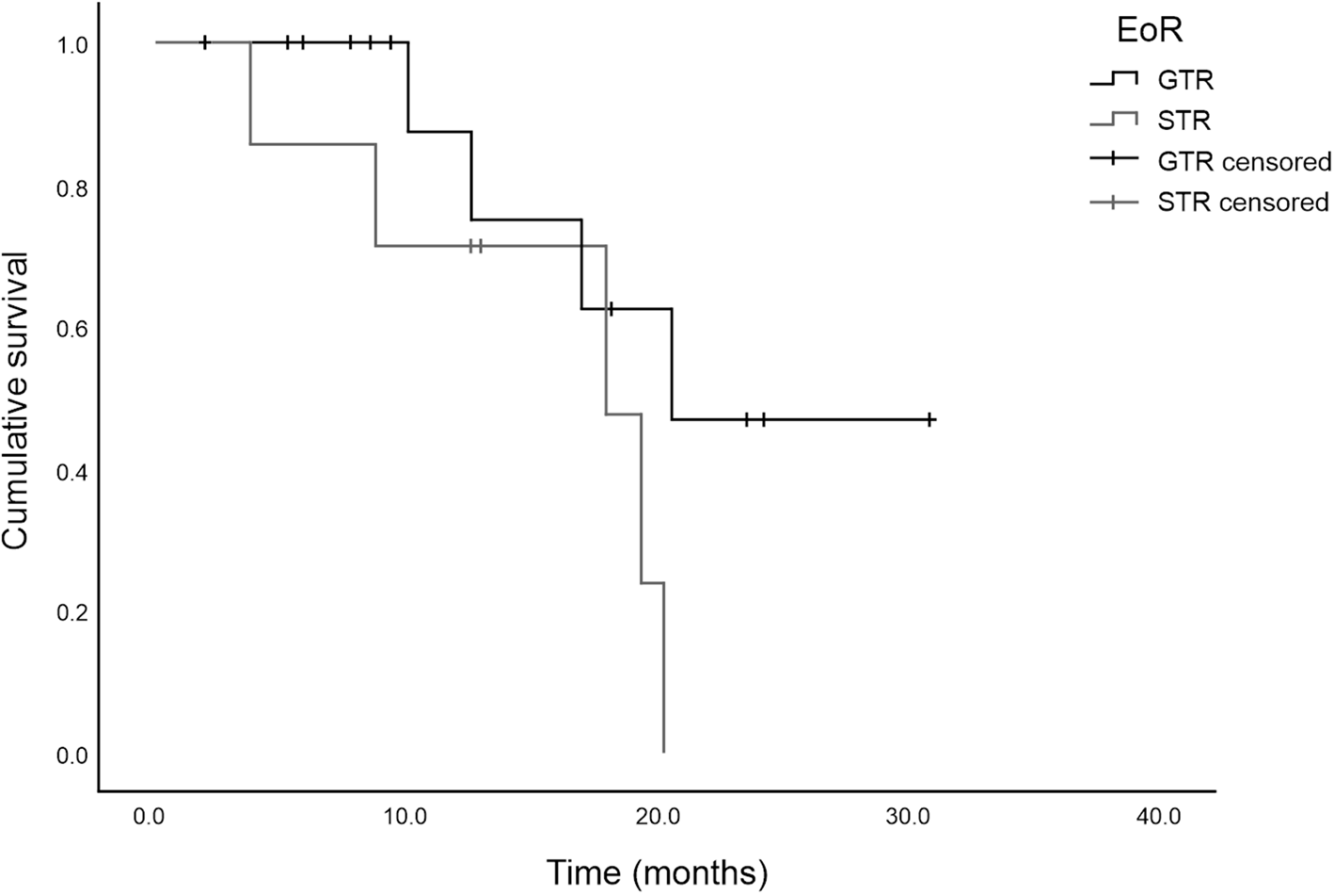
Kaplan–Meier survival curve for all 21 patients stratified by extent of tumor resection.

Data on clinical and survival outcomes are summarized in Table 3. During the median observational period of 12.6 (range 2.0–30.9) months, including 2 patients being operated on within the preceding 6 months to the final evaluation, 9 patients died from glioblastoma after 17.0 (3.8–20.6) months, while the other 12 patients were still alive after 9.4 (2.0–30.9) months. Of these, 3 patients suffering from glioblastoma presented with progressive disease after a median progression-free survival of 12.5 (7.6–19) months. Thus, the calculated estimated median OS was 19.4 months for both, the entire patient cohort as well as the cohort of exclusively glioblastoma patients (Fig. 2 and supplementary figure s1, respectively).

**Table 3:** Clinical and survival outcomes.

Outcomes	Value
Overall follow-up period, months, median (range)	12.6 (2.0–30.9)
**Survival**	
6-mo survival rate, n (%)	18 (94.7%)*
12-mo survival rate, n (%)	12 (80.0% )^†^
Alive at last follow-up, n (%)	12 (57.1%)
Disease progression in patients alive	3 (23.1%)
**Clinical**	
KPS before discharge (t2), (n = 16)	90 (50–100)
KPS after 5.3 months (t3), (n = 12)	100 (70–100)

*n = 19; in 2 of the 21 patients the 6-months follow-up have not yet been reached.

^†^n = 15; in 6 of the 21 patients the 12-months follow-up have not yet been reached.

All patients’ median KPS score was 100 prior to surgery and 90 before discharge. On an individual level, 4 patients had an improved KPS score following surgery, whereas a worsened KPS score was noted in 6 patients. Of the latter, 5 patients suffered from transient SMA syndrome and one patient experienced aggravation of pre-existing aboulia, improving during the postoperative hospital stay. At the time of long-term neurocognitive re-evaluation, those patients still alive, being followed-up for at least 6 months and consenting to neurocognitive re-evaluation, presented a median KPS score of 100. Most importantly, only 2 of these 9 patients presented with slight neurological deficits at this time; both patients suffered from mild gait disturbances. Of those 2 patients being observed less than 6 months, one patient had minor speech initiation difficulty after surgery, while the other patient was unimpaired.

### Neurocognitive outcome

**Figure 3:**
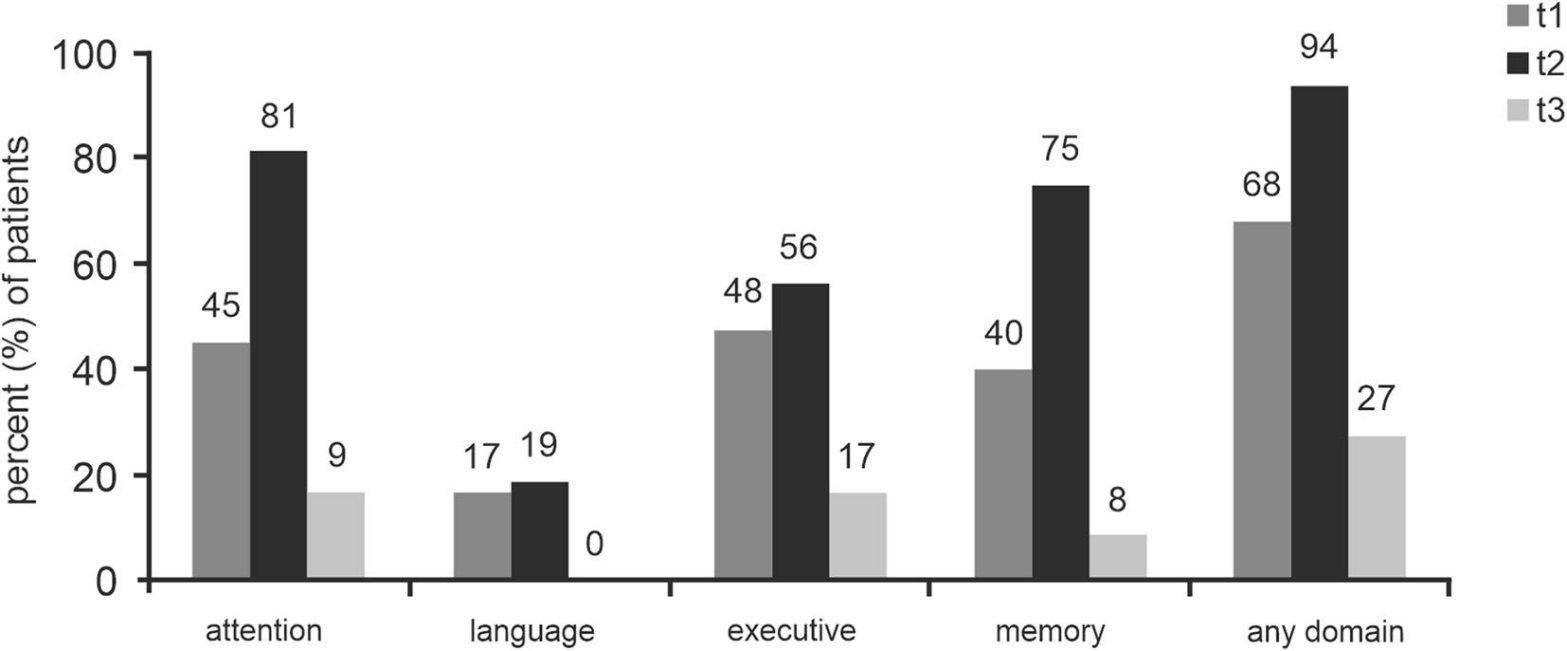
Domains, for which normative data were available. Percentage of patients impaired per domain, considered as a decrease in a Z-score of more than 1.5 SD, at time points t1 (prior to surgery), t2 (before hospital discharge) and t3 (at long-term follow-up).

Considering a decrease in a Z-score of more than 1.5 SD as cognitive decline, the percentage of patients impaired per domain at the different time points is depicted in Fig. 3 and listed in Table 4. After surgery, at t2, the percentage of impaired patients increased for all domains, with a significant increment in attention (*p* = 0.027). Most importantly, after the median follow-up period of 5.3 months, at t3, neurocognitive dysfunction was observed in a smaller number of patients in any domain, reaching significance in the domains attention, executive functioning and memory (*p *= 0.01, *p* = 0.034 and *p* < 0.001, respectively).

**Table 4:** Patients’ neurocognitive function in the different domains over time.

Cognitive domain	Percentage of impaired patients (− 1.5SD)			*P* value T1 versus T2	*P* value T2 versus T3	*P* value T1 versus T3
	t1	t2	t3			
**Referenced to normative data**						
Attention	45.0	81.3	16.7	0.027*	0.001*	0.102
Language	16.7	18.8	0.0	0.874	0.112	0.136
Executive functioning	47.6	56.3	16.7	0.603	0.034*	0.075
Memory	40.0	75.0	8.3	0.036	< 0.001*	0.054
**No normative data available**						
Orientation	15.8	18.8	8.3	0.817	0.436	0.546
Visuospatial functioning	36.8	43.8	8.3	0.678	0.040*	0.077
Figurative memory	52.6	68.8	50.0	0.332	0.315	0.886
Bimanual coordination	57.9	68.8	41.7	0.508	0.152	0.379
Object recognition	63.2	68.8	58.3	0.728	0.569	0.788
Depression	21.1	30.8	0.0	0.533	0.044*	0.102
Anxiety	27.8	30.8	9.1	0.856	0.193	0.228

∗ *P* value < .05, defining significant deviation.

**Figure 4:**
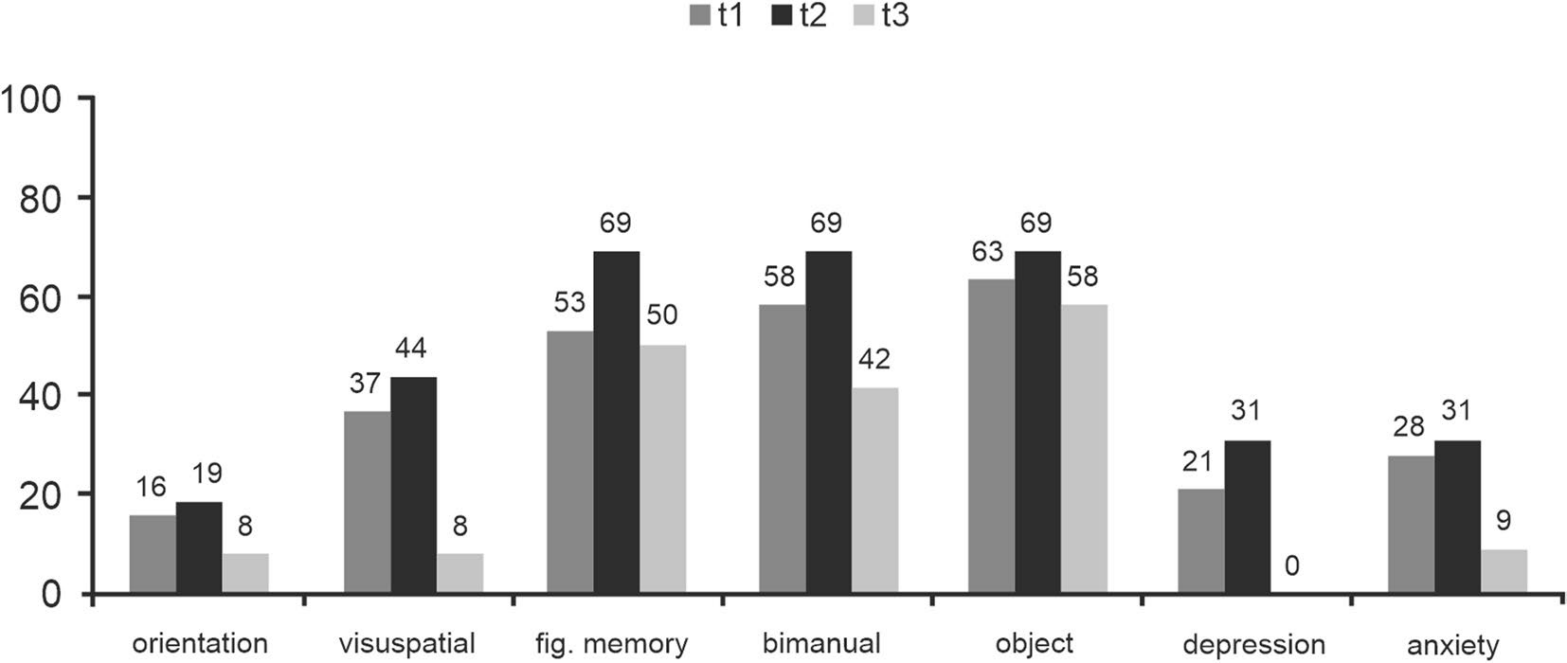
Domains, for which no normative data were available. Percentage of patients impaired per domain, considered as a decrease in a Z-score of more than 1.5 SD, at time points t1 (prior to surgery), t2 (before hospital discharge) and t3 (at long-term follow-up).

Those neurocognitive tests, for which no normative data were available, provided similar results (Fig. 4); the number of patients with worsened neurocognitive performances increased in all domains between time points t1 and t2. At t3, the number of impaired patients had decreased in all domains compared to both, the pre- and postoperative neurocognitive assessment, with visuospatial functioning improving significantly during the postoperative course (*p* = 0.040).

## Discussion

### Key findings

To our knowledge, this is the first study investigating neurocognition in patients who underwent brain tumor resection within the corpus callosum. The key findings of this investigation are that (1) tumor invasion of the corpus callosum did not result in shortened OS compared to the according literature on tumors not invading the corpus callosum, if tumor resection was performed; (2) after resection of tumors with corpus callosum invasion neurocognition only declined within the first postoperative days, whereas (3) after at least 3.1 months, patients’ performances had improved dramatically in every neurocognitive domain.

### Interpretation in the context of surgery

The number of reports on tumor resection within the corpus callosum is scarce. One of the first retrospective studies was provided by Steltzer et al. in 1997, comparing OS in patients resected for high grade glioma with and without corpus callosum involvement and showing that median OS decreased from 105 to 57 weeks, if the corpus callosum was involved^15^. However, a correlation of OS and EoR was not provided until 2015, when Chen et al. investigated the impact of corpus callosum involvement on EoR, progression-free survival and OS^11^. Analyzing data of 22 high grade glioma patients among 33 patients with corpus callosum invasion, they observed longer median progression-free survival (ranging from 3.8 to 10.0 months) and longer median OS, (ranging from 7.1 to 20.9 months) in patients who underwent higher EoR, with an EoR > 85% being of statistical significance.

In the present study complete tumor resection could be achieved in 15 patients, and in 6 patients median EoR was 97.7%, with median residual tumor volume of 2.1 cm^3^. Estimated median OS was 19.4 months for both, the entire cohort as well as the cohort of exclusively glioblastoma patients, being in line with results of key trials on high grade glioma patients. Thus, in glioblastoma patients, the combination of radiotherapy and temozolomide was reported to result in OS of 14.6 months in the EORTC 26981/22981 trial^16^, in OS of 16.6 months in the RTOG 0525 trial^17^, and, by adding tumor-treating fields in the EF-14 trial, in OS of 20.5 months^18^. Of note, no stratification according to MGMT promotor methylation and IDH mutation status was performed in these trials, allowing for a comparison with the hereby presented data on patients with tumors of different molecular features. However, recent literature on the resection of butterfly glioblastoma reported on shorter median OS; the largest according series of patients was published by Chaichana et al. in 2014, and they observed median OS of 6.4 months in 29 patients who underwent surgical resection of butterfly glioblastoma^9^. The difference in OS to the present study might not only be explained by the fact, that we included both, butterfly glioblastoma and tumors invading the corpus callosum, but also by the administration of adjuvant radiotherapy and/ or chemotherapy to only 66% and 55% of their patients compared to radiochemotherapy to 81% of our patients. Another series by Burks et al. reported on longer median OS in 27 butterfly high grade glioma patients, with median OS coming up to 15 months^19^, whereas in a series published by Dayani et al., median OS was 14.1 months^10^. Most importantly, patients who underwent biopsy of butterfly glioma experienced significantly shortened OS, ranging from 1.5 to 4.2 months^9,10,12,20^. Among our presented study cohort only one patient experienced similar short OS: the patient was 76 years old, suffered from huge left frontal glioblastoma and was the only one presenting slight aboulia, that temporary worsened after subtotal tumor resection and improved until hospital discharge. Rapid early tumor progression hampered further adjuvant treatment, and she died 3.8 months after surgery. Another 8 patients, all undergoing operation for glioblastoma, died after a median of 17.5 months.

Whether aboulia might be associated with shortened OS is speculative. However, none of the other 21 patients experienced aboulia or postoperative callosal disconnection syndrome. Burks et al. reported on immediate postoperative aboulia in 11 (44%) of 25 patients who underwent standard tumor resection and in 1 (7%) of 15 patients who underwent a cingulum-sparing technique with awake subcortical mapping^19^. In the present study, all surgical procedures were conducted in patients under general anesthesia using subcortical motor mapping and continuous somatosensory evoked potential monitoring. In 3 patients with strictly callosal situated lesions we chose an interhemispheric approach, while in all other patients a transcortical frontal approach along the axis of the tumor was performed. In case of tumor invasion surgical resection comprised the cingulate gyrus accordingly, otherwise, the cingulum was spared. Interestingly, none of the 11 patients who underwent tumor resection within the cingulate gyrus presented aboulia or akinetic mutism, but slight SMA-syndrome with transient contralateral weakness was observed in 5 of these patients due to tumor resection extending into the posterior superior frontal gyrus. Although the small number of patients does not allow for a meaningful conclusion, we presume that (1) the selection of patients with a preoperative relatively high KPS score, (2) with tumors mainly situated in the frontal lobe, and (3) the conduction of surgery with intraoperative neurophysiological monitoring and mapping might have been responsible for the relatively low number of postoperative neurological deficits observed in our cohort of patients.

### Considerations on neurocognition

Neurocognitive impairment is frequent in brain tumor patients. Depending on tumor aggressiveness, tumor size, location, and genetic characteristics impairments in neurocognitive function have been observed in up to 83% of treatment-naïve glioma patients^21–24^. However, data on neurocognitive outcome after glioma surgery are less frequent and provide heterogeneous results. An effort to summarize these data has been made only recently by Ng et al., including 11 studies on a total of 313 patients, of whom 45% suffered from high grade and 55% of low grade glioma^25^. In sum, and despite the heterogeneity of data, an improvement of patients’ performances was observed as early as within the first days after surgery in all cognitive domains, except for executive function that sustainably declined after surgery.

Data of the present study contrast with these results. Prior to surgery neurocognitive dysfunction was observed in no more than 63.2% of our patients, although all but one patient suffered from high grade glioma of large volume. During the postoperative course, all cognitive functions worsened and the proportion of impaired patients increased in every domain, whereas at long-term follow-up evaluation, a clear improvement of neurocognitive dysfunction was observed. At this time point, language was impaired in none of the patients, and less than a fifth of patients were impaired in attention, executive functioning, memory, orientation or visuospatial functioning. Only the rate of impairment on figurative memory, bimanual coordination and object recognition remained elevated, affecting 50%, 41.7% and 58% of patients, respectively. Indeed, postoperative white matter integrity through corpus callosum might explain these sustained cognitive impairments. Nevertheless, the interhemispheric information transfer necessary for bimanual coordination and tactile recognition has been attributed to the posterior body and the splenium of the corpus callosum^26,27^, being affected in only two and one patient, respectively, in the present study cohort by surgical tumor resection. Sustained impairment on figurative memory, on the other hand, seems more consistent with lesions in anterior parts of the corpus callosum. Since successful visual memory encoding engages both, episodic and semantic memory, an interhemispheric transfer between the right occipito-temporal connections responsible for pure visual information and mostly left-lateralized frontal and temporal semantic hub areas is required^28,29^. This might explain the relatively high percentage of sustained impairment of visual memory following the disconnection of left lateralized semantic areas and right lateralized areas needed for visuo-construction, whereas at the same time verbal memory and language, both less depending on interhemispheric communication, improved over time. In order to investigate whether ongoing impairments of visual memory depend on interhemispheric connections future studies might use tests applying non-semantic coding items like abstract signs. However, with exception of these functions critically depending on interhemispheric transfer, in total, patients’ neurocognitive abilities dramatically improved within several weeks after surgery, consequently improving patients’ everyday functioning and their quality of life.

### Limitations

The major limitation of our study is the incompleteness of follow-up neurocognitive data sets. Due to patients’ refusal to undergo further postoperative cognitive evaluation (n = 5), death (n = 3) or study inclusion within the 6 months preceding the final evaluation (n = 1) a longitudinal follow-up was only possible in 12 patients. Thus, the heterogeneity of patient cohorts at different time points may have biased presented results. Moreover, it is likely that only patients with relatively good clinical performance biased our results. On the one hand, only patients with tumors that were amenable to a great extent of tumor resection were included into our study; on the other hand, patients who were willing and able to undergo long-term follow-up cognitive evaluation might have possibly caused an overestimation of clinical and neurocognitive results. Nonetheless, surgery-related outcome measures such as EoR and OS were assessed in all patients, enabling unbiased comparison to the relevant literature and strengthening the concept of surgical resection of tumors with corpus callosum invasion.

Further evaluations in a bigger cohort of patients is mandatory, in order to allow for further meaningful correlations of clinical, surgical, histopathological and cognitive data. Future findings may encourage surgeons to perform resection rather than biopsy in patients with huge corpus callosum invading lesions, positively impacting both, OS and quality of life in these patients.

## Conclusion

The results of the present study suggest benefit from complete or subtotal resection of huge tumors invading the corpus callosum. No long-term decline but dramatic improvements in patients’ OS and neurocognitive functioning was observed, providing evidence that in patients with gliomas infiltrating the corpus callosum, who preoperatively present in rather good neurological state, the benefit of tumor resection might outweigh morbidity.

## Supplementary information

Supplementary information.

## Data Availability

Data on individual patients’ neurocognitive testing are available on request.

## References

[1] Bloom JS, Hynd GW (2005). The role of the corpus callosum in interhemispheric transfer of information: Excitation or inhibition?. Neuropsychol. Rev..

[2] Gazzaniga MS (2000). Cerebral specialization and interhemispheric communication: Does the corpus callosum enable the human condition?. Brain.

[3] Hopkins WD, Rilling JK (2000). A comparative MRI study of the relationship between neuroanatomical asymmetry and interhemispheric connectivity in primates: Implication for the evolution of functional asymmetries. Behav. Neurosci..

[4] Morton BE (2013). Behavioral laterality of the brain: Support for the binary construct of hemisity. Front. Psychol..

[5] Seymour SE, Reuter-Lorenz PA, Gazzaniga MS (1994). The disconnection syndrome. Basic findings reaffirmed. Brain.

[6] Sperry, R. W., Gazzaniga, M. S. & Bogen, J. E. in *Disorders of Speech, Perception and Symbolic Behavior. Handbook of Clinical Neurology.* pp. 273–290 (North-Holland, Amstedam, 1969).

[7] Broekman ML (2018). Multidimensional communication in the microenvirons of glioblastoma. Nat. Rev. Neurol..

[8] Quail DF, Joyce JA (2017). The microenvironmental landscape of brain tumors. Cancer Cell.

[9] Chaichana KL (2014). The butterfly effect on glioblastoma: Is volumetric extent of resection more effective than biopsy for these tumors?. J. Neurooncol..

[10] Dayani F (2018). Safety and outcomes of resection of butterfly glioblastoma. Neurosurg. Focus.

[11] Chen KT (2015). Corpus callosum involvement and postoperative outcomes of patients with gliomas. J. Neurooncol..

[12] Dziurzynski K (2012). Butterfly glioblastomas: A retrospective review and qualitative assessment of outcomes. J. Neurooncol..

[13] Hendriks EJ (2018). Linking late cognitive outcome with glioma surgery location using resection cavity maps. Hum. Brain Mapp..

[14] Lezak MD (1995). 1995 Neuropsychological Assessment.

[15] Steltzer KJ, Sauve KI, Spence AM, Griffin TW, Berger MS (1997). Corpus callosum involvement as a prognostic factor for patients with high-grade astrocytoma. Int. J. Radiat. Oncol. Biol. Phys..

[16] Stupp R (2005). Radiotherapy plus concomitant and adjuvant temozolomide for glioblastoma. N. Engl. J. Med..

[17] Chinot OL (2014). Bevacizumab plus radiotherapy-temozolomide for newly diagnosed glioblastoma. N. Engl. J. Med..

[18] Stupp R (2015). Maintenance therapy with tumor-treating fields plus temozolomide vs temozolomide alone for glioblastoma: A randomized clinical trial. JAMA.

[19] Burks JD (2017). A method for safely resecting anterior butterfly gliomas: The surgical anatomy of the default mode network and the relevance of its preservation. J. Neurosurg..

[20] Opoku-Darko M, Amuah JE, Kelly JJP (2018). Surgical resection of anterior and posterior butterfly glioblastoma. World Neurosurg..

[21] Boone M, Roussel M, Chauffert B, Le Gars D, Godefroy O (2016). Prevalence and profile of cognitive impairment in adult glioma: A sensitivity analysis. J. Neurooncol..

[22] Noll KR (2019). Monitoring of neurocognitive function in the care of patients with brain tumors. Curr. Treat. Options Neurol..

[23] vanKessel E (2019). Tumor-related neurocognitive dysfunction in patients with diffuse glioma: A retrospective cohort study prior to antitumor treatment. Neurooncol. Pract..

[24] Wefel JS, Noll KR, Rao G, Cahill DP (2016). Neurocognitive function varies by IDH1 genetic mutation status in patients with malignant glioma prior to surgical resection. Neuro Oncol..

[25] Ng JCH (2019). Effects of surgery on neurocognitive function in patients with glioma: A meta-analysis of immediate post-operative and long-term follow-up neurocognitive outcomes. J. Neurooncol..

[26] Fabri M (2005). Contribution of posterior corpus callosum to the interhemispheric transfer of tactile information. Brain Res. Cogn. Brain Res..

[27] Gooijers J, Swinnen SP (2014). Interactions between brain structure and behavior: The corpus callosum and bimanual coordination. Neurosci. Biobehav. Rev..

[28] Nenert R, Allendorfer JB, Szaflarski JP (2014). A model for visual memory encoding. PLoS ONE.

[29] Tomasello R, Garagnani M, Wennekers T, Pulvermuller F (2018). A neurobiologically constrained cortex model of semantic grounding with spiking neurons and brain-like connectivity. Front. Comput. Neurosci..

[30] Tombaugh TN, Kozak J, Rees L (1999). Normative data stratified by age and education for two measures of verbal fluency: FAS and animal naming. Arch. Clin. Neuropsychol..

[31] Kaplan E, G. H., Weintraum S.  (1983). Boston Naming Test.

[32] Aschenbrenner S, Tucha O, Lange KW (2000). Regensburger Wortflüssigkeits-Test.

[33] Helmstaedter C, Lendt M, Lux S (2001). Verbaler LErn- und Merkfähigkeitstest (VLMT).

[34] Fountoulakis KN (2011). The standardised copy of pentagons test. Ann. Gen. Psychiatry.

[35] Luria, A. R. *Higher Cortical Functions in Man*(1980).

[36] Peschke, V. *Handanweisung Burgauer Bedside-Screening* (Selbstverlag PSYDAT, 2004).

[37] Cockrell JR, Folstein MF (1988). Mini-Mental State Examination (MMSE). Psychopharmacol. Bull..

[38] Zigmond AS, Snaith RP (1983). The hospital anxiety and depression scale. Acta Psychiatr. Scand..

