# Supplementary material for: Benefits of glioma resection in the corpus callosum
Source: Sci Rep. 2020 Oct 6;10:16630. doi: 10.1038/s41598-020-73928-x (PMC7538917; doi:10.1038/s41598-020-73928-x)

Marie-Therese Forster, MD, MBA ^1,2^*, Marion Behrens^3^*, Irina Lortz^1^, Nadine Conradi, M.Sc.^3^, Christian Senft, MD^1,2^, Martin Voss, MD^2,4^, Maximilian Rauch, MD^2,5^, Volker Seifert, MD^1,2^

**Benefits of glioma resection in the corpus callosum**

1. Department of Neurosurgery, Goethe University Hospital, Schleusenweg 2-16, 60528 Frankfurt am Main, Germany

2. University Cancer Center Frankfurt (UCT), Theodor Stern Kai 7, 60590 Goethe University Hospital, Frankfurt, Germany

3. Department of Neurology, Goethe University Hospital, Schleusenweg 2-16, 60528 Frankfurt am Main, Germany

4. Dr. Senckenberg Institute of Neurooncology, Goethe University Hospital, Schleusenweg 2-16, 60528 Frankfurt am Main, Germany

5. Department of Neuroradiology, Goethe University Hospital, Schleusenweg 2-16, 60528 Frankfurt am Main, Germany

* These authors have contributed equally to this article.

**Supplementary Figure s1.** Kaplan-Meier survival curve exclusively for glioblastoma patients (n=17) stratified by extent of tumor resection.


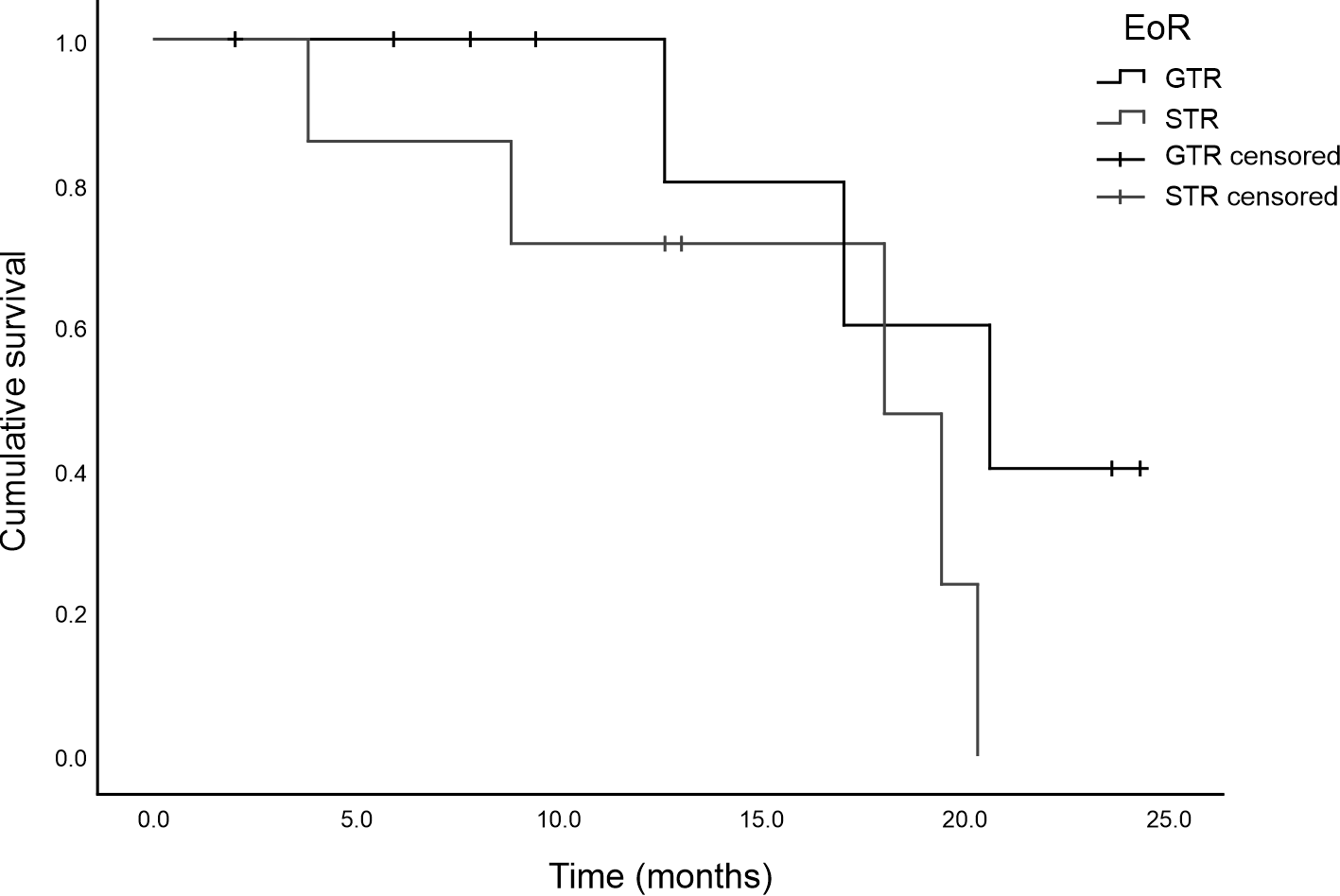

Supplement: Supplementary file 1 — Supplementary information. [file 41598_2020_73928_MOESM1_ESM.docx]
